# Rare cardiac sequelae of a hump-nosed viper bite

**DOI:** 10.1186/s13104-015-1426-z

**Published:** 2015-09-14

**Authors:** Sharmila Thillainathan, Dilani Priyangika, Indika Marasinghe, Karunayokiny Kanapathippillai, Gayani Premawansa

**Affiliations:** Colombo North Teaching Hospital, Ragama, Sri Lanka

**Keywords:** Myocardial infarction, Brain injury, Hump-nosed viper

## Abstract

**Background:**

The hump-nosed pit viper (*Hypnale hypnale*) is the commonest cause for venomous snakebites in Sri Lanka. Previously, it was thought to cause only local envenomation. However recently, several systemic effects and even mortality has been reported. Along with other snakes, such as the Indian cobra (*Naja naja*), the common krait (*Bungarus caeruleus*), the Russell’s viper (*Daboia russelii*) and the saw-scaled viper (*Echis carinatus*), the hump-nosed viper is now also considered capable of causing lethal envenomation. Unlike other snake species, the systemic manifestations occurring through the bite of a hump-nosed viper, such as acute renal failure, thrombotic microangiopathy etc are rare and unpredictable.

**Case presentation:**

A 49-year-old Sri Lankan Tamil male presented with a hump-nosed viper bite, which had resulted in a cardiac arrest within half an hour of envenomation. On arrival to the Emergency Treatment Unit, he was unconscious and without spontaneous breathing. Electrocardiography monitoring revealed ST elevation in leads II, III and aVF with reciprocal changes in leads I and aVL—indicating inferior wall infarction—as well as atrial fibrillation. Glasgow Coma Scale was 7/15, which indicated severe brain injury and electroencephalogram on day 10 revealed a low amplitude pattern compatible with diffuse brain damage.

**Conclusion:**

This case describes an authenticated case of myocardial infarction in a 49-year-old male following envenomation by a hump-nosed viper in Sri Lanka. This systemic effect of this viper’s bite has not previously been described in the literature. This case report is intended to increase the vigilance for myocardial infarction following hump-nosed viper envenomation.

## Background

The hump-nosed pit viper (genus *Hypnale*) is the commonest cause for venomous snakebites in Sri Lanka [1]. Previously, it was thought to cause only local envenomation, but of late, several systemic effects and even mortality has been reported [2–4]. Along with the Indian cobra (*Naja naja*), common krait (*Bungarus caeruleus*), Russell’s viper (*Daboia russelii*) and saw-scaled viper (*Echis carinatus*), the hump-nosed viper is now also considered as capable of causing lethal envenomation [2]. Coagulopathy, acute kidney injury and other organ injury/involvement are known systemic envenomation effects. Unlike other snake species, the systemic manifestations of a hump-nosed viper bite are rare and unpredictable [3]. We present a case of a hump-nosed viper bite, resulting in diffuse brain injury following a transient inferior myocardial infarction and atrial fibrillation, which occurred within half an hour of envenomation, which has not previously been described in literature. Acute myocardial infarction is a recognized phenomenon in other species of vipers [5, 6] but not in the hump-nosed viper.

## Case presentation

**Fig. 1:**
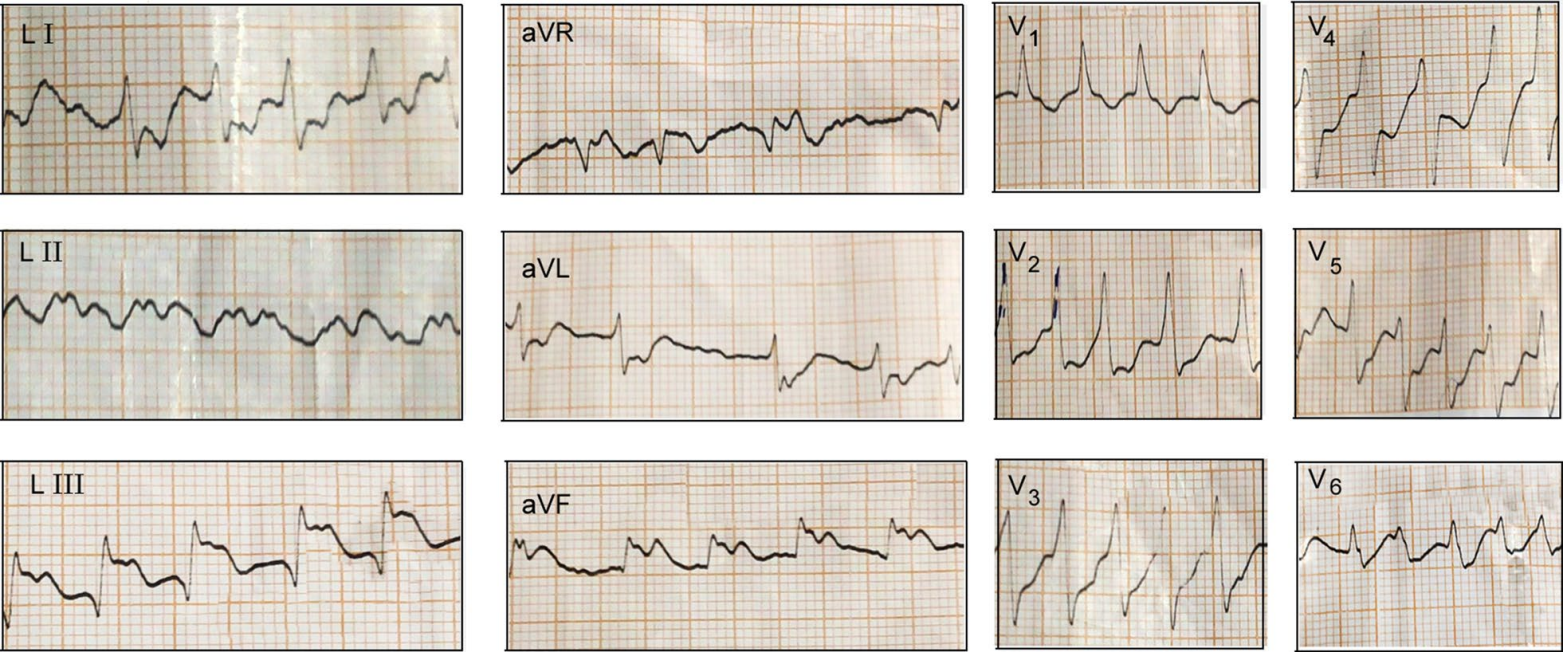
Electrocardiogram done on admission showed ST elevation in inferior leads as well as atrial fibrillation

A 49-year-old Sri Lankan Tamil male suffered a snake bite to his left foot while working in the garden. The snake had been killed and brought in to hospital for identification purposes. It was subsequently identified as a hump-nosed viper. On the way to hospital, the patient developed severe retrosternal chest pain and became unresponsive to stimuli, however did not receive cardiopulmonary resuscitation (CPR) during transport. On arrival to the Emergency Treatment Unit (ETU), he was unconscious with a Glasgow Coma Scale (GCS) of 3/15, and did not have spontaneous respiration. Electrocardiography (ECG) revealed ST elevation in leads II, III and aVF with reciprocal changes in leads I and aVL, suggestive of an inferior wall infarction (Fig. 1). The ECG also showed atrial fibrillation with a heart rate of 132 min^−1^. His blood pressure at time of presentation to ETU was 68/40 mmHg. Immediate cardiopulmonary resuscitation was commenced and he was urgently intubated, and synchronized electrical cardioversion was performed with a 100 joule initially, followed by 200 J. This led to the return of spontaneous circulation and converted his rhythm back to sinus rhythm at 110 min^−1^, with a blood pressure of 122/80 mmHg. Fang marks were seen over the dorsal aspect of the first toe in left foot, with associated swelling and localized bleeding but no signs of necrosis. Twenty-minute whole blood clotting time (20-WBCT) was prolonged indicating ongoing coagulopathy. A subsequent 12-lead ECG repeated 90 min later revealed resolved ECG changes other than T-wave inversion in lead III.

His past medical/surgical history included diabetes mellitus for which he was taking metformin for the last 2 years. He did not have any hypertension or ischemic heart disease in the past.

Intensive care was provided. 20-WBCT persisted to be prolonged for another 48 h. GCS improved to 7/15. His full blood count showed marked neutrophil predominance (87 %), leukocytosis with white blood cell count of 26.5 × 10^9^/L, haemoglobin of 110 g/L, haematocrit of 44 % and the platelet count was 101 × 10^9^/L. Coagulation profile revealed prolonged prothrombin time of 23 s (control 12 s) with international normalized ratio (INR) of 1.9. Renal profile was normal with serum Na 140 mmol/L, K 3.72 mmol/L and serum creatinine 0.9 mg/dL. C-reactive protein was high (160 mg/L). He developed haematuria on the first day with urine red blood cell count at 30–40/hpf. This resolved by the third day of admission. Initial blood film showed presence of a few schistocytes indicating early micro-angiopathic haemolytic anaemia (MAHA), which resolved in consequent films following fresh frozen plasma therapy. Thrombolysis, anticoagulation and anti-platelets were not considered due to the presence of haematuria and coagulopathy. The cardiac enzyme troponin I assay was marginally elevated. The electroencephalogram (EEG) performed on day 10 revealed low amplitude pattern compatible with diffuse brain damage. Imaging, which included a non-contrast computed tomography scan of the brain and a transthoracic echocardiogram to assess the heart, were normal.

He was extubated after a tracheostomy on day 8. Over the next 2 weeks, his GCS showed improvement from 7/15 to 10/15. Almost a month after the incident, he was discharged home with arrangements for rehabilitation. According to the Glasgow–Pittsburgh Cerebral Performance Categories (CPC) Scale, he persisted to be in cerebral performance category 3 on discharge, as well on follow-up at 6 months [7].

## Discussion

Hump-nosed viper snakebites are common in southern India and Sri Lanka. Bites by hump-nosed pit vipers were previously underestimated to only result in local envenomation; however, debilitating fatal systemic envenomation is a recognized phenomenon now, with more than one organ system being affected [2–4].

Cardiac manifestations are relatively less common following snake bite envenomation. Fatal deaths caused by cardiac arrest following myocardial infarction have been reported following Russell viper envenomation in Sri Lanka [5], but not following hump-nosed viper bites. In 1999, a study by Seneviratne et al. analyzed cardiac symptoms, ECG changes and troponin T levels of 32 patients, and reported that myocardial damage did not seem to be an important feature of Sri Lankan hump-nosed viper bites [8]. In our patient, initial ECG following his cardiac arrest revealed ST elevation in inferior leads of II, III and aVF with reciprocal changes associated with cardiac arrhythmia to suggest acute inferior wall myocardial infarction. A positive troponin level further supports it; however, prolonged CPR may also contribute to the elevated troponin levels.

The exact mechanism of myocardial infarction following snakebite envenomation is unclear and potential causes are suggested as follows: (1) toxin or electrolyte-mediated cardiac arrhythmia leading to coronary hypoperfusion and demand ischemia. (2) Toxin mediated myocardial necrosis or transient myocardial effects leading to decreased cardiac output [9]. (3) Coronary thrombus due to haematological venom effects [10]. (4) Coronary vasospasm due to cardio-toxic effect [6]. (5) Cardiac arrest secondary to other causes such as vascular collapse due to venom and/or cytokine effects, and hypovolemic shock due to vascular permeability and/or bleeding.

A study on the enzymatic and toxicological effects of *Hypnale hypnale* venom by Tan et al. revealed that in addition to the haemorrhagic and necrotic properties, *H. hypnale* venom also showed potent pro-coagulant or thrombin-like enzymes—such as phospholipase A2, thrombin-like enzyme, proteases, l-aminoacidoxidase and hyaluronidase—suggesting potential coronary thrombosis following envenomation [10].

Coronary artery spasm is considered as an important etiological cause for myocardial infarction following snakebites, which could be due to the cardio-toxic effect of the toxin itself or due to the adrenergic surge following the bite [6]. Considering the retrosternal chest pain followed by the sudden cardiac arrest within a short interval following the envenomation, coronary spasm seems to be a possible causative factor. This is further evidenced in our case as the ECG changes were transient and echocardiogram did not reveal any residual ischemic changes. (Coronary angiogram was not performed due to the condition of the patient at time of admission). A direct toxic effect of viper venom on myocardial tissue may lead to shock which might have been the result of the venom’s cardiotoxic effect which, besides damaging the myocardium, may trigger cardiac arrhythmia as was seen in our patient [9].

Hyperkalemia following an acute renal failure is another cause for sudden cardiac arrests following snakebites. Nevertheless, it is unlikely in our patient as his serum potassium level and renal function remained within normal range throughout. Consumption coagulopathy leading to a hypercoagulable state resulting in thrombus formation in a coronary vessel(s) may be a possible aetiology in this case.

Brain injury persists to be the leading cause of disability after cardiac arrest, despite seminal advances in intensive care and cardiovascular therapy over the past several decades [11]. Delayed presentation to the emergency facility and prolonged CPR lasting more than 10 min may be contributing factors for the diffuse brain injury seen in this patient. Direct effect of venom on brain cells may also play a part. Long-term care of these patients can be challenging and requires a great deal of medical resources and expenses.

We are currently unable to predict which of this hump-nosed viper envenomation will have severe systemic manifestations and clinicians need to be vigilant regarding this possibility. Also, the only available anti-venom (raised against krait, cobra, Russell’s viper and saw-scaled viper venoms) is ineffective against the hump-nosed viper, and carries a high risk of anaphylactic reactions [4]. Considering the several fatal systemic manifestations of a hump-nosed viper bite, urgent need for appropriate anti-venom is of utmost importance.

## Conclusion

This unique case provides evidence for cardiac arrest following a hump-nosed viper bite, highlighting the need to be vigilant of rare systemic manifestations and the urgent need for invention with appropriate anti-venom for hump-nosed viper envenomation.

## Consent

Written informed consent was obtained from the patients next of kin for publication of this Case Report and any accompanying images. A copy of the written consent is available for review by the Editor-in-Chief of this journal.

## Abbreviations

CPR: cardio pulmonary resuscitation
ETU: Emergency Treatment Unit
GCS: Glasgow Coma Scale
ECG: electrocardiography
20 WBCT: twenty minute whole blood clotting time
INR: international normalized ratio
MAHA: micro angiopathic haemolytic anaemia
EEG: electroencephalogram
CPC: Cerebral Performance Categories Scale (CPC)
